# Clinical students’ perception of educational environment in a Nigerian university: a mixed method study

**DOI:** 10.1186/s12909-024-05734-2

**Published:** 2024-07-04

**Authors:** Aderonke O. Akinpelu, Olufemi O. Oyewole, Nse Odunaiya, Adesola C. Odole, Jesupelumi P. Olley

**Affiliations:** 1 Physiotherapy Department, University of Ibadan, Ibadan, Nigeria; 2 Department of Physiotherapy, Olabisi Onabanjo University Teaching Hospital, Sagamu, Nigeria; 3 College of Health Sciences, University of Kwazulu-Natal, Private Bag X54001, Durban, South Africa

**Keywords:** Clinical students, Perception of learning environment, University of Ibadan, Nigeria

## Abstract

**Background:**

Learning environment (LE) research has been given priority in higher education institutions globally because of its influence on learning processes and outcomes. Although studies reporting the perceptions of health science students about LE in Nigeria are available, none have compared the perceptions of students from different health professions. Therefore, this study aimed to assess final-year clinical students’ perceptions of their LE from four programs (dentistry, medicine, nursing, and physiotherapy) and compared their LE perceptions.

**Methods:**

This study adopted a cross-sectional study design using a mixed method approach. The quantitative survey involved all the final-year clinical students at the University of Ibadan, and they completed the Dundee Ready Education Environment Measure (DREEM) questionnaire. The qualitative aspect involved 24 consenting students in four focus group discussions.

**Results:**

A total of 214 out of 223 copies of the DREEM questionnaire were duly completed and returned, yielding 96.0% response rate. The participants’ mean age was 24 ± 2.3 years (ranged between 22 and 25 years, *p* = 0.001). The mean DREEM scores of the students from the four programs ranged between 119.68 ± 18.02 and 147.65 ± 15.89 out of a maximum of 200, interpreted as more positive than negative perceptions of LE. Physiotherapy students’ DREEM score was significantly higher than those of medical, dental, and nursing students (*p* < 0.001). The DREEM scores of other students did not differ significantly (*p* > 0.05). Dental and medical students had similar positive perceptions. The qualitative aspect revealed that the students had positive perceptions of their teachers’ knowledge base and self-acquisition of knowledge but negative perceptions of their teachers’ communication skills, infrastructural facilities, lecturer-student relationships, and hostel accommodations.

**Conclusion:**

Although the survey indicated that these clinical students had more positive than negative perceptions of their learning environment, the qualitative aspect of the study revealed many challenges that the students were confronted with. The clinical students’ perception of their learning environment could be improved if the university authorities would address these challenges.

**Supplementary Information:**

The online version contains supplementary material available at 10.1186/s12909-024-05734-2.

## Background

Assessing the learning environment has been given priority attention in higher education institutions globally. This is because the learning environment not only influences learning processes but also affects learning outcomes [1, 2]. A positive perception of the learning environment was associated with specific learning processes and improved overall learning experience through better motivation and engagement [1]. A positive perception of the learning environment has also been shown to influence future career planning among nursing students [3]. A positive perception of the learning environment leads to subjective happiness, well-being, and satisfaction [4, 5]. Since stress has been shown to correlate negatively with the learning environment, among clinical students, evaluating students’ perceptions of their learning environment enables school authorities to provide them with supportive facilities through numerous orientation programs, counseling units, academic advice, and workshops [5, 6]. Furthermore, assessing the learning environment can facilitate changes in the educational environment to improve learning behavior and outcomes [7]. Students’ perceptions of their learning environment provide them with a louder voice through which they can share their experiences in school [8].

There seemed to be a gap in deep insight into the learning environment among health professional students, especially in Nigeria, on how learning outcomes could be enhanced. A mixed-method research approach was suggested to provide this insight, which virtually has not been previously studied in Nigeria [9]. These deep insights were necessary because some of the programs were due for curricular review at the institution of interest. The present study was designed to fill this gap in knowledge among clinical students using a mixed-method research approach. This study provides in-depth insight into the perceptions of clinical students about their learning environment to enhance learning outcomes.

Learning is the process whereby knowledge is created through the transformation of experience [10]. The learning environment is generally understood as a combination of factors that affect the teaching-learning process and learners’ perspectives, such as interpersonal relationships, teaching methodology, infrastructure, availability of facilities, cultural compliance with the university curriculum, and everything that happens within the classroom, department, faculty, or university [6]. However, advances in technology have influenced learning, and the learning environment can be virtual, online or remote. On the other hand, a clinical learning environment involves everything that surrounds the students, including the clinical setting, the staff, and the patients. It has been described as a complex social context of interactive forces within a practical setting that influences students’ clinical and professional learning outcomes while being closely monitored by educators [11]. High-quality clinical placement provides students with opportunities for skill development, socialization into the profession, and a bridge between academic and workplace training [12].

Students’ perceptions of their learning environment have been reported among health sciences students globally. Most of the related studies used the Dundee Ready Education Environment Measure (DREEM) questionnaire to assess the learning environment among medical students [13–17], dental students [18–21], nursing students [6, 22, 23], physiotherapy students [24–26], podiatry students [27], and veterinary students [28, 29]. These studies reported more positive than negative perceptions of the learning environment among health sciences students. Generally, preclinical students reported more positive perceptions than did students in the clinical phase [2, 23, 30–32]. Few studies have attempted to compare students’ perceptions of the learning environment across programs such as medicine, nursing and midwifery, physiotherapy, dentistry, and public health; nursing students reported a greater positive perception of the learning environment than medical students did [31, 33, 34].

To gain more insight into students’ learning environment, several studies have employed qualitative methods to explore their perceptions [35–38]. The broad theme from these studies includes “the perceived journey to become a professional; the perceived structure and culture; and the perceived relationship with the supervisor”, which were positive [38]. Further themes include “the gap of transferring formal teaching from lab skills to clinical placement, learning self-leading procedural skills in clinical settings, and students’ dissatisfaction with patients’ vulnerability” [37] and “feedback processes, assessments and grading, and tutor interactions” [36]. Other themes were “context of learning, the context of teachers, the context of student’s perception of their academic skills, the context of atmosphere and context of social life” [35]. Using a mixed-methods approach may provide better insight into clinical students’ learning environment than employing either qualitative or quantitative methods.

Most studies reporting learning environment perceptions among health sciences students from Nigeria [25, 32, 39–44] collected data using the DREEM questionnaire and reported more positive than negative perceptions. Studies on LE perceptions from Nigeria that utilized a mixed-method approach appear uncommon. The aim of this study was therefore to determine the learning environment perceptions of final year students in four undergraduate courses at the College of Medicine, University of Ibadan, using a mixed method approach and compared the four programs’ LE.

## Methods

### Study context

The University of Ibadan started as the University College, Ibadan, which was founded in 1948. The Faculty of Medicine was one of the three faculties of the university at its inception; others were the Faculties of Arts and Science. During the 1980/81 session, the Faculty of Medicine was upgraded to a collegiate status with three faculties (Faculties of Basic Medical Sciences, Clinical Sciences and Dentistry, and Pharmacy). Currently, there are four faculties in the college, and these are the Faculties of Basic Medical Sciences, Clinical Sciences, Dentistry, and Public Health; the Faculty of Pharmacy moved out of the college to become an independent faculty. The College of Medicine runs five undergraduate clinical degree courses, Bachelor of Medicine and Surgery (MBBS), Bachelor of Dental Surgery (BDS), Bachelor of Nursing Science (BNS), Bachelor of Physiotherapy (B. Physio), and Bachelor of Medical Laboratory Science (BMLS). The MBBS and BDS each run a six-year program, which comprises two semesters of basic science courses, three semesters of basic medical science courses, and seven semesters of clinical science courses. The other undergraduate clinical programs in the college run five-year courses, comprising two semesters of basic science courses, two to three semesters of basic medical science courses, and five to six semesters of clinical science courses. Students from the college attend most classes together in basic science and basic medical science courses. As at the time this study was conducted in 2016, BMLS students were not yet in their final year. Among the four clinical programs involved in the study, 223 final-year students were enrolled [dentistry (28), medicine and surgery (135), nursing (37), and physiotherapy (23)]. The mode of instruction in all the programs was English. The curricula of some of the programs were due for review.

The College of Medicine is domiciled on the premises of the University College Hospital, Ibadan, which is approximately 15 km from the main university campus. The students received basic science and basic medical courses on the main university campus. The College of Medicine has a hostel that could accommodate only approximately 60% of the students at the time of this study. The Nursing science students were accommodated in halls of residence on the main university campus.

### Design

The study adopted a cross-sectional study design using a mixed method approach to capture more in-depth information on the subject matter. Both quantitative and qualitative data were collected at the same time and analyzed separately. The quantitative survey aspect of the study involved all (223) final-year dental, medical, nursing, and physiotherapy students at the College of Medicine, University of Ibadan. We chose final-year clinical students because they had passed through both the preclinical and clinical phases of their programs. The qualitative aspect of the study involved 24 consenting final-year students; six from each of the four programs who were purposively selected.

The DREEM questionnaire was used to collect information on the students’ perceptions of their learning environment. The DREEM is a validated and reliable inventory that was originally designed in English [45]. It has been cross-culturally adapted to many other languages, including Polish, Swedish, and Greek [46–48]. It has been used in many studies of healthcare education worldwide [17–19, 22, 23, 42, 43]. The reported DREEM overall Cronbach’s alpha ranged between 0.89 and 0.93, while the domains ranged between 0.55 and 0.86 [41, 48, 49]. The DREEM comprises 50 statements relating to a range of topics directly relevant to the learning environment. Items on the DREEM are in the form of statements relating to the participant’s learning environment (e.g., “I am encouraged to participate in class”); these statements are rated via a 5-point Likert scale, where 4 = strongly agree, 3 = agree, 2 = uncertain, 1 = disagree, and 0 = strongly disagree. The DREEM has five domains: students’ perceptions of learning (SPOL, 12 items), students’ perceptions of lecturers (SPOT, 11 items), students’ academic self-perceptions (SASP, 8 items), students’ perceptions of the atmosphere (SPOA, 12 items) and students’ social self-perception (SSSP, 7 items). The maximum obtainable score on the DREEM is 200, which indicates the ideal educational learning environment as perceived by the student. A score of zero is the minimum and would be a very worrying result. The overall DREEM scores were interpreted as follows: 0–50, very poor; 51–100, plenty of problems; 101–150, more positive than negative; and 151–200, excellent [45]. The English version of the DREEM questionnaire was administered to all consenting students after being duly informed about the aim of the study.

Six participants from each clinical program were involved in four focus group discussions. Participants were assured of confidentiality and were advised to feel free to discuss, as their responses would not be used against them. A focus group discussion guide adapted from a previous study [35] and based broadly on the perception of learning, the perception of teachers, student self-academic perception, the perception of learning atmosphere, and the perception of social life was used during the focus group discussion (see appendix 1). The questions served as a guide only as the moderator asked other questions and used comments from the participants to encourage discussion. Prompts were used to clarify participants’ responses and to elicit more complete responses to the questions asked. This interview focus group discussion guide served to maintain uniformity in the topics that were explored during the interviews. A tape recorder was used to record the experiences of the students involved in the focus group discussion, and there were two note takers. The moderators of the focus group discussion were part of the researchers and lecturers from the Department of Physiotherapy, University of Ibadan, who were experienced in conducting focus group discussions. One of the researchers and an assistant took notes. The nature, purpose, and procedure of the research were explained to the participants in detail by the researcher, and informed consent was obtained before the commencement of the study. All four focus group discussions took place in the new Physiotherapy Department Building, University College Hospital, Ibadan between August and September 2016 in the afternoon hours. The duration of FGDs ranged between 100 and 140 min.

### Data analysis

Descriptive analysis of percentages, mean and standard deviation was used to summarize the sociodemographic data of the participants. For quantitative data, ANOVA was used to assess the overall perceptions and the domains of students’ learning environment across the four programs, and a Tukey’s HSD post hoc test was used to show within group comparisons. IBM Statistical Product and Service Solutions (IBM-SPSS) software version 25 was used. The level of statistical significance was determined by a *p*-value of < 0.05. Two independent transcriptionists transcribed the recordings and made use of field notes during the process. Deductive thematic analysis was carried out for the data obtained from the focus group discussions by the authors. The six steps developed by Braun and Clarke were employed for the thematic analysis. These include becoming familiar with the data, coding, searching for themes, reviewing themes, defining and naming themes, and producing the report [50]. The themes were generated based on the domains of the DREEM questionnaire. Three of the authors manually generated codes assigned to the based-on theme of DREEM, and common themes were described based on frequency.

## Results

Table 1 presents the characteristics of the quantitative survey participants. A total of 214 out of 223 copies of the DREEM questionnaire were duly completed and returned by the final year clinical students, yielding 96.0% of the responses. There were more female than male participants (ratio 1.4:1, *p* = 0.001). The highest proportion of the participants came from medicine and surgery (60.75%), while participants from other programs had between 10.75% and 15.42% participants. There was significant difference in age across the programs (F = 13.59, *P* = 0.001). Six participants from each of the four programs participated in the focus group discussion comprising 66.7% (16) females.

**Table 1:** Characteristics of the quantitative survey participants

Program	Gender					Age		
	Both gender	Male	Female	χ2	*P*			
	*n* (%)	*n* (%)	*n* (%)			mean ± SD	F	*P*
Dentistry	28 (13.08)	15 (7.01)	13 (6.07)	18.600	0.001	25.0 ± 2.5	13.59	0.001
Medicine & Surgery	130 (60.75)	58 (27.10)	72 (33.64)			23.0 ± 2.3		
Nursing	33 (15.42)	3 (1.40)	30 (14.02)			25.0 ± 2.5		
Physiotherapy	23 (10.75)	13 (6.07)	10 (4.67)			22.0 ± 2.0		
All students	214 (100)	89 (41.59)	125 (58.41)			23.8 ± 1.3		

**Fig. 1:**
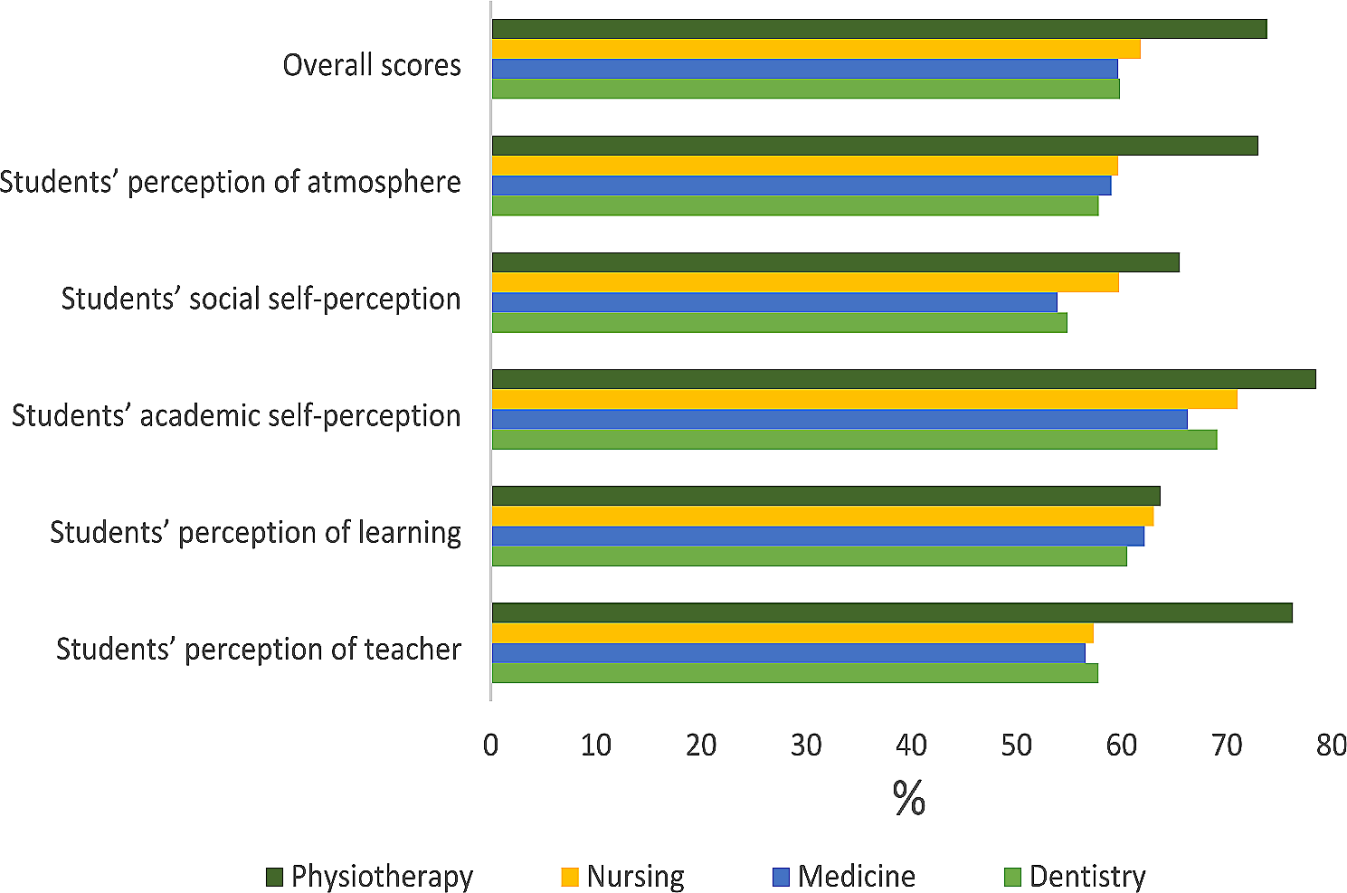
Percentage DREEM scores by domain *The percentage (%) for each scale is based on the maximum value attainable for that scale

A total DREEM score of 119.68 ± 18.02 and 119.36 ± 23.36 (*p* = 0.946) revealed that dentistry and medicine students, respectively, had more positive perceptions of their learning environment than negative perceptions. The SASP had the highest percentage of domain possible scores (66.31-69.09%), while the SSSP had the lowest percentage of domain possible scores (53.89-54.86%). The nursing students had an overall DREEM score of 123.61 ± 22.49, and their SASP scores had the highest percentage of domain possible scores (71.03%), while their SPOT scores had the lowest percentage of domain possible scores (57.36%). The physiotherapy students had an overall DREEM score of 147.65 ± 15.89; their SASP score was the highest (78.53%), while their SPOL score was the lowest (63.69%). The physiotherapy students’ overall DREEM scores were significantly greater than those of the dental, medical, and nursing students (Table 2; Fig. 1; F = 11.111; *P* < 0.001). The medicine and surgery students had the lowest overall DREEM scores. The same pattern was observed across domains of DREEM (*p* < 0.001) except for students’ perception of learning (*p* = 0.801) for the four programs. The within group comparison of the programs is shown in Table 3. The physiotherapy students’ overall scores of the DREEM questionnaire were significantly greater than those of the dental, medical, and nursing students (*p* = 0.001). There was no significant difference in overall DREEM scores between the nursing, dental and medical students (*p* > 0.05).

**Table 2:** Comparison of DREEM scores by domain

Domain	Dentistry	Medicine	Nursing	Physiotherapy	F	*P*
Students’ perception of teacher (44)*	25.43 ± 4.48	24.88 ± 5.74	25.24 ± 5.88	33.57 ± 4.27	16.762	< 0.001
Students’ perception of learning (48)*	29.04 ± 5.04	29.85 ± 6.43	30.27 ± 5.92	30.57 ± 4.07	0.334	0.801
Students’ academic self-perception (32)*	22.11 ± 6.85	21.22 ± 4.02	22.73 ± 3.27	25.13 ± 2.83	5.830	0.001
Students’ social self-perception (28)*	15.36 ± 2.15	15.09 ± 4.09	17.73 ± 3.21	18.35 ± 2.50	8.705	< 0.001
Students’ perception of atmosphere (48)*	27.75 ± 5.47	28.33 ± 6.51	28.64 ± 8.89	35.04 ± 5.07	7.063	< 0.001
Overall score (200)*	119.68 ± 18.02	119.36 ± 23.36	123.61 ± 22.49	147.65 ± 15.89	11.111	< 0.001

*Maximum obtainable score

**Table 3:** Within group comparison of final-year responses from dentistry, medicine, nursing, and physiotherapy students across the domains of DREEM (post hoc analysis)

Programs compared	Domain															Overall score		
	Students’ perception of teacher			Students’ perception of learning			Students’ academic self-perception			Students’ social self-perception			Students’ perception of atmosphere					
	MD	95%CI	*p*	MD	95%CI	*P*	MD	95%CI	*P*	MD	95%CI	*P*	MD	95%CI	*P*	MD	95%CI	*P*
Physiotherapy vs. Dentistry	8.14	5.66 - 10.62	0.001	1.53	-1.09 - 4.15	0.246	3.02	-0.05 - 6.09	0.054	2.99	1.68 - 4.30	0.001	7.29	4.30 10.28	0.001	27.97	18.31 - 37.6	0.001
Physiotherapy vs. Medicine	8.69	6.21 - 11.17	0.001	0.72	-2.03 - 3.47	0.605	3.91	2.18 - 5.64	0.001	3.26	1.52 - 5.00	0.001	6.71	3.89 9.54	0.001	28.29	18.27 - 38.31	0.001
physiotherapy vs. Nursing	8.33	5.45 - 11.21	0.001	0.30	-2.56 - 3.16	0.834	2.40	0.71 - 4.09	0.006	0.62	-0.98 2.22	0.441	6.40	2.28 10.52	0.003	24.04	13.11 - 34.97	0.001
Nursing vs. Medicine	0.36	-1.86 - 2.58	0.749	0.42	-2.01 - 2.86	0.734	1.51	0.02 - 3.00	0.048	2.64	1.13 - 4.15	0.001	0.31	-2.40 - 3.02	0.822	4.25	-4.67 - 13.18	0.349
Nursing vs. Dentistry	-0.19	-2.91 - 2.53	0.889	1.23	-1.62 - 4.08	0.391	0.62	-2.07 - 3.31	0.646	2.37	0.94 - 3.80	0.002	0.89	-2.98 - 4.78	0.647	3.93	-6.64 - 14.50	0.460
Dentistry vs. Medicine	0.55	-1.73 - 2.83	0.635	-0.81	-3.37 - 1.75	0.532	0.89	-1.02 - 2.80	0.358	0.27	-1.30 1.84	0.735	-0.58	-3.19 - 2.03	0.661	0.32	-8.95 - 9.59	0.946

MD: Mean difference; CI: Confidence interval; P: *P*-value

Twenty-four clinical students participated in the four focus group discussions. Each participant was given the opportunity to contribute to each theme and was allowed to build on previous comments by other participants or move on if they had different comments other than what previous participants had stated. Seven themes were identified that revolved around the five domains of DREEM. The qualitative results (verbatim spoken words of participants) are presented in italics.

### Theme one: perception of curriculum/course content

Eighteen final-year clinical students contributed to the theme perception of the curriculum and course content. Most physiotherapy students generally believed that their curriculum and course content were good but that some of their courses were bulky, while a few were scant. Below are some of the excerpts from the qualitative data:

*“Approximately 30% of the lecture materials are bulky (e.g., Intensive Care Physiotherapy and Disorders of Lymph and Blood Vessels“***(P1, male).**

*“Courses like Gerontology had very few outlines and should thus be reviewed as there was truly nothing much to do“***(P2, female)**.

The medical students also shared similar views with physiotherapy student. Most perceived their courses as very bulky, and students were expected to know so much in very little time.

*“The new MBBS curriculum is very good; however, it was created for a perfect system that does not exist yet, and we are expected to work like robots”. “The medical curriculum was designed as though it was meant for robots with no feelings or life outside medical school. As a result, students boycott a lot of processes and only come to sign their clinical booklets, thereby coining out names such as MBBS*, i.e., *Master of Booklets, Bachelor of Signatures”*. **(M6, male)**.

*“The plan was to make it student-centered, but it is not. The curriculum says that learning should be self-directed, for example, but if I do not even understand the little, I am being taught in class; then, there is little that I can learn on my own. For example, I am supposed to be taught with mannequins, but I have not even seen them before, and I will be tested on them in the exams. Other times the equipment is faulty, and too many students are waiting to observe a certain procedure like biopsy due to pile-up of students from industrial actions“***(M1, female).**

Almost all the dentistry students agreed with the views of medical students that the curriculum is very demanding because there is so much to cover in very little time.

*“The new curriculum places a lot of demand on us because the time has been shortened yet the course content and workload have increased, and exams come more frequently except in final year where the exam has been split into two. However, we are more knowledgeable as the curriculum places more emphasis on practice rather than theory“***(D5, male).**

All the Nursing students expressed similar views like other students in the other programs that the curriculum is very wide, and little time is allocated to it.

*“Our courses are very bulky, especially at the 400th level, yet we have very little time. Additionally, the curriculum does not afford us the opportunity to resit our exams in the same academic year, and all courses are compulsory apart from the pass mark being 50, whether elective or not“***(N5, female).**

### Theme two: perception of teaching methods and long-term effects of learning

Sixteen clinical students contributed to the theme. All the physiotherapy students agreed that most of their lectures were student-centered, but many were deficient in case scenarios and discussions that enable learning to have long-term effects. They also reported that sometimes the information given in class conflicts with the information they receive from the internet and that there is sometimes an overlap of information when two lecturers teach a course.

*“85% of the learning is student-centered, and it helps me to adequately prepare for my profession, as I can express myself freely“***(P4, male).**

*“It is student-centered since I can argue with my lecturer based on what I understand, and he/she agrees to go read about it; then, we come back to discuss”. “There is usually an overlap of course content when two lecturers take a particular course; thus, I prefer having just one lecturer handle the course“***(P6, female).**

*“No adequate time, outlines are rushed through thereby placing pressure on students to read and understand before exams“***(P1, male).**

*“Even though self-directed learning is being incorporated, there is too much contradictory information gotten on the internet in comparison to what is being taught in class. Self-directed learning should also be checked, and consistent and case studies should be related to course content”. “Only courses where case scenarios were used enable me to learn in the long-term. Also reading at my own pace makes understanding easier, but since I must prepare for exams in a short time, then I just rush through my reading without clearly understanding it“***(P5, female).**

*“Case scenarios are effective, but they should be well time-tabled, and group presentations should be supervised by lecturers“***(P2, female).**

Most medical students felt contra wise that their lecturers were student-centered. They felt that they were just rolling blindly, as their teachers did not give them adequate information on what they should know in class, expecting them to check everything online. They identified that the lecturers also sometimes abuse them verbally during ward rounds.

*“As a result of inadequate information available to both students and teachers, massive failure is inevitable since the students do not know the standards by which they will be judged till after the exams. Students are just rolling blindly, as some laws and bylaws can suddenly be used against them”. “The term self-directed learning has been abused as lecturers expect you to know everything on your own, whereas I cannot google every single thing coupled with the fact that I cannot use my phone in the class. Additionally, the Odeku library has been under construction for the past two years (it does not even look like any work is going on there), so books are not accessible, and access to information is limited*” **(M5, female).**

*“People have emotional and psychological breakdowns because they cannot cope with the workload. People who lose loved ones are still expected to write major tests and examinations and nobody cares; even lecturers tell us that they expect some students to have a mental breakdown and that truly, it happens. We are thus abused verbally on ward rounds and students who cannot handle the trauma and insults breakdown or go into substance use“***(M1, female).**

*“Monitoring is key, but the procedures should also be engaging and participatory, as not all lecturers are good at engaging students while doing what they are doing. Some lecturers even insult us, ignore us, or send us out as though we are an inconvenience to them“***(M6, male)**.

“*However, not all the postings are bad as psychiatry is a very lovable one and the doctors in O and G (Obstetrics and Gynecology) are very friendly. plastic surgeons are very friendly too such that one is tempted to ask them why they are not getting angry“***(M1, female).**

However, few dental students asserted that their lecturers know what they are supposed to teach; however, they are not student-centered and do not relay their knowledge in a student-friendly manner.

*“Our learning is not student-centered, and it is mostly short-term. We do not have the opportunity to make mistakes and learn from them yet so much is expected from us in a short time. The lecturers are also intimidating, and some derive joy in failing students”* (**D5, male**).

The nursing students have additional views that methods of teaching should be more practical-intensive than just theory-based.

*“I just read to pass except I have residual knowledge about something. The curriculum is too wide; thus, only 50% of our learning is long-term, and we rush our lectures toward exams. We also do not have feedback from our learning, and self-directed learning is abused*” **(N6, female).**

*“Learning sticks more with practical classes. We do not have enough clinical instructors as those available do not supervise our clinical postings; moreover, there are discrepancies in the ideal and the clinics“***(N3, female).**

*“Strike actions affect our calendar, and the department does not update our rotations properly as we do the same thing every time. I think ward rounds and block postings should be included in the curriculum, and we need professional nurses to teach us rather than other lecturers teaching us abstractly“***(N7, female).**

*“Some lecturers relate their experiences to learning, thus making learning easy, while some cannot communicate effectively. Some just read out their slides. We should be told the standard, but the teaching should still be contextualized”. “Only a few lecturers make use of feedback mechanisms or bring in their experiences“***(N4, female).**

### Theme three: perception of lecturers’ knowledgebase

Most participants from the four programs agreed that their lecturers are knowledgeable, but they have varying ways of communicating their knowledge to students.

*“Approximately 85–90% of the lecturers are knowledgeable, but some do not have a good delivery system. Additionally, schools of thought are very different and can be confusing“***(P2, female).**

*“Based on clarity of information gotten by me from the lecturers, I will say that they are quite knowledgeable except when they take courses that are outside their specialty“***(P3, male).**

*“Our lecturers are knowledgeable but cannot truly communicate effectively. Oral pathology is very good at updating slides, whereas restorative departments are very poor at communicating, and some lecturers do not have organized lecture notes. Some lecturers just teach based on experience rather than knowledgebase”* (**D4, female**).

### Theme four: perception of learning at the preclinical and clinical phases of the study

Eleven clinical students contributed to the theme. Learning during the preclinical phase of study was generally difficult, and the physiotherapy students thought that they would have learned better if they were taught separately from students in other programs, especially medicine and dentistry.

*“In the preclinical years, physiology practical classes were not enjoyable at all as we were too many and limited by availability of equipment, space, and time. the environment was distracting, as many of our courses were clashing, and we did not even know about some courses until weeks before examinations; however, we did better in anatomy when we were separated from medical students”***(P3, male).**

*“Even if we are taught separately, the fact that we are being taught by medical doctors rather than physiotherapists limits what we can learn that will be beneficial to use”* (**P1, male).**

The clinical training of the dental students is not a relief from what they experienced in their preclinical years.

*“Preclinical years were stressful because the classes were rowdy, and the lecturers used medical terms we did not understand. In clinical school, there is a trend of agama syndrome (bending all the time to greet every lecturer”* (**D2, male**).

*“Clinical years have been very disappointing, verbal abuse is the order of the day, students are demoralized. Equipment is not always available, the workload is much, and we are fatigued”* (**D4, female**).

*“The students’ questions are automatically turned into assignments or bad-mouthed by the lecturer, so students do not want to ask questions. Classes are not comfortable; a technical person is needed to avoid distractions for the student doing the technical work, and a schedule of the lectures held should be noted”* (**D6, male**).

However, medical students felt that clinical postings are often affected by industrial actions, and individuals sometimes struggle with rushing between classes and clinical postings.

*“I feel we do not get the best. Our clinical postings involve groups and certain factors, such as industrial actions, heavy workloads both on clinicians and students, nonavailability of information, and short durations of disruptive learning. There are usually clashes in course objectives, assignments, and classes. Nevertheless, we are still expected to read up so many things within a little amount of time“***(M3, female).**

*“Some clinical postings are more organized than others because we know what we ought to do at the right time. The most organized posting is Pediatrics, O & G is somewhat organized (we only learn when there is a registrar willing to teach), while Radiology is the least organized“***(M4, female).**

*“The large number of students at a time is a major deterrent to very good learning, as we have to come in batches, but at the end of the day, everybody is fatigued from standing for several hours, and you dare not leave because if any information is given in your absence, you might never get it again”* (**M5, female).**

### Theme five: perceptions of lecturer-student relationships and feedback mechanisms

Eleven clinical students contributed to the theme. Most physiotherapy students had a cordial relationship with their teachers, and some of the lecturers adopted a feedback mechanism in class.

*“We have a very cordial relationship with our lecturers. use of pictures, videos, models, objects, and other illustrations are very good ways of communicating“***(P5, female).**

*“Asking specific questions helps to request feedback appropriately, but this is adopted by only approximately 60% of the lecturers“***(P2, female).**

However, the dental students had contrary views stated that they do not have a strong cordial relationship with most of their lecturers, as they find them intimidating.

*“We are fearful about relating with some of our lecturers, as there is a boss-servant relationship, even among the junior lecturers and their senior colleagues”* (**D1, female**).

*“Most of our lecturers are not readily available or approachable; hence, we cannot share our problems with them”.***(D3, male)**.

Most of the nursing students shared similar views with dental students, reported not being free to discuss anything with their teachers.

*“We are intimidated by our lecturers as they make us look stupid whenever we interact with them, and nobody wants to be a scapegoat”* (**N2, female**).

*“40% of our lecturers are student-centered, most are authoritative, and very few admit that they do not know something. I believe there should be an attitude upgrade once you are educated, and that should affect your social relationship”* (**N1, female**).

*“Our lecturers are not readily available; their workload is high; thus, they are very edgy and ultimately transfer the aggression to us and for those pursuing their own careers too we have to suffer for it”* (**N5, female**).

### Theme six: perception of the learning environment (infrastructure/facilities)

Sixteen clinical students contributed to the theme. The classrooms in the physiotherapy department are conducive with functioning air conditioners, but the lack of a functioning library is a major limitation.

*“The classrooms are generally conducive, but we do not have a functioning library, and individual practical classes are necessary after theory classes, and at times, the time for practical is being used up by the other lecturer taking the same course“***(P5, female).**

The learning environment consists of everything in and out of the classroom that affects learning in one way or the other, and the students do not have ready access to those things. However, most students in all the programs felt that they do not have access to most of the equipment that facilitates learning, and they can only see most of them for the first-time during examinations.

*“Equipment to be used for learning is locked up safely only to be brought out for the very first time in the exam, and of course, I would not know what to do with them, and so much is expected of me as a graduate of the University of Ibadan in the outside world. Even simple instruments such as tourniquets might not be identifiable by students under exam conditions, and examiners laugh at students during examination, thus demoralizing the student“***(M2, female).**

*“We do not have much equipment; we use our money to purchase expensive instruments, and we are forced to replace faulty ones. We also lack a functioning library and no access to e-books; thus, we depend only on our lecture notes. Our chairs are terrible, afternoon classes are uncomfortable as air-conditioners do not function“***(D4, female).**

The stress of accommodation can deter students’ academic performance. Most students in all the programs desired improvement in accommodation facilities.

*“Crossing over from preclinical years is very challenging because there is no accommodation for us. Refrigerators and other appliances that make life easier are taken from us, yet the basis for comparing us with those on the main campus is not fair“***(D5, male)**.

*“At times, we do not have water in the hostel; the porters expect us to do their work for them. The security level is zero, and students’ opinions do not matter; rather, they use us for business, especially concerning food and cafeteria”* (**D2, male**).

The nursing students desired that they would be accommodated properly within the social premises, especially in clinical areas.

*“In terms of accommodation, there is no priority for us, yet we stay late in school and have to get to the clinic early. We should be accommodated in the clinical areas“***(N1, female).**

*“There is no water or light in the hostels, and the accommodation process is very stressful. Particularly in the female hostels, as they keep demanding ridiculous documents such as admission letters, the number of students in a room is large, thus contributing to emotional stress; the porters are very rude and favor some students only because of personal gain“***(N4, female).**

### Theme seven: perception of the effects of clinical training on students’ social life

All the participants in the four programs agreed that their social life is adversely affected by their academic studies, as they hardly have time to attend social gatherings outside their department.

*“Physiotherapy is a very selfish course because I finish classes by 6 pm every day beginning at 8 am. It has thus affected my social life“***(P6, female).**

*“We do not have time for parties; we do not have friends outside our department, but we are willing to sacrifice our socials so we can graduate on time. Even our lecturers do not expect us to have a social life“***(D2, male).**

*“We do not get to socialize even among ourselves, and we have had issues with our friends because we failed to attend their social gatherings. We cannot even take sick leave or go to eat during lectures; hence, we rarely have friends outside our department”***(N2, female).**

*“It is actually relative, but for me, I have had to turn down so many offers because I would rather be reading my books as failure to do that would be at my own detriment“***(P5, female).**

## Discussion

The quantitative survey indicated that the overall mean DREEM scores for participants’ perceptions of their learning environment for the four programs fell within the range interpreted as more positive than negative. Some of these negative experiences revealed by focus group discussions bordered on social life, infrastructures, and lecturer-student relationships. The students’ perception of their learning environment may therefore be described as fair. Generally, clinical students from Nigerian universities expressed more positive than negative perceptions of the learning environment [25, 40, 42, 43]. The mean DREEM scores in our study were similar to those of students from some Nigerian universities and elsewhere [25, 33, 41, 43] but higher than those of students from other Nigerian universities [39, 42], as well as those of students from India and Iran [16, 31]. However, this proportion was lower than that reported for Australian [10], Sri Lankan clinical students [14], and a few Nigerian universities’ clinical students [40, 51]. The higher DREEM scores in these studies may suggest greater innovation in student-centered learning than in the present study [10]. Most students in the focus group discussions, especially from medicine and dentistry programs affirmed that their learning was not student-centered. Student-centered learning has been shown to improve the perception of the learning environment [14]. According to the percentages of the transformed DREEM scores, the domain of the DREEM that was rated highest by students in the four programs was the Students’ Self-Academic Perception (SASP). This implies that the students perceive themselves as having a high ability to learn. Previous studies reported similar findings among clinical students [25, 39–41]. The dental and medical students in the present study had the same rating pattern for the five domains of the DREEM; the students’ perception of learning (SPOL) was the second highest rating, and the students’ social self-perception (SSSP) was the lowest (fifth). Nursing and physiotherapy students also rated the SSSP third and fourth, respectively. This may be because clinical students’ workloads are generally high, and this does not give them time to socialize. This is particularly true for dental and medical students (as suggested by anecdotal evidence), who rarely go on vacation once they reach the clinical phase of their studies. The lower rating of SSSP domain scores was similar to previous observations in Nigeria and elsewhere [14, 40, 43, 51]. A lower rating of SSSP has been attributed to greater stress among students [40, 43]. The focus group discussions corroborated this further and provided more insight into how the students perceived that their social life was negatively affected. The students from the four programs agreed that they do not have time to socialize because of their academic workload. This should be taken into consideration when reviewing the curriculum of the programs that are due for review. The new curriculum should accommodate relaxation and socialization; in particular, the focus should be on breaks between hours of didactic learning.

The physiotherapy students’ rating of Students Perception of Teachers (SPOT) was second of the five domains and the best of the four groups, while the rating by the nursing students was lowest (fifth) and that of the medical and dental students was fourth. This varied perception/experience was buttressed by the results of focus group discussion from the four programs. This may imply that, at the College of Medicine, University of Ibadan, clinical students perceive the lecturer-student relationship as poor, and there is a need for the authorities at the College of Medicine, University of Ibadan, to improve this important aspect of the learning environment. This finding was in tandem with those of previous studies reporting varied perceptions of the domains of DREEM among clinical students [10, 31, 33, 52]. These differences have been attributed to the extent of curriculum success among different programs or faculty profiles [31, 33]. The focus group discussions shed more light on this negative lecturer-student relationship. The students affirmed that most lectures were not student-centered and did maintain boss-servant relationships; thus, they were intimidated and afraid to approach or relate to lecturers. This negative lecturer–student relationship may hinder free discussion with lecturers even if they have problems that warrant doing so. It has been suggested that a positive lecturer–student relationship encourages students to learn better and achieve more [35]. In reviewing the curricula of clinical science programs, the authorities of the College of Medicine of the University of Ibadan may create departmental counselors for each program to improve lecturer–student relationships. A similar study from the same institution suggested the provision of departmental counselors rather than a central support system. This approach would improve the awareness of such support and enhance its utilization [25].

The dental, medical, and physiotherapy students all rated their percentage of students’ perception of atmosphere (SPOA) as third out of the five domains, while the nursing students rated it fourth. This might imply that the perceived learning atmosphere was not optimal, and that adequate attention is required to improve it. This observation was similar to those of previous studies in which clinical students’ SPOA was rated third or fourth [14, 40, 51]. Under the theme ‘perception of learning environment’, the students indicated a poor state of infrastructure. Students claimed that they had inadequate classroom facilities, lacked the necessary equipment for practical training and lacked a functional library. Except for physiotherapy students, many of the classrooms had no air conditioners or good chairs, thus making learning especially difficult, especially in the afternoon when it is generally hot. Some of these negative experiences that focus on inadequate infrastructures were noted earlier by a previous study [35]. In addition, the main library of the College of Medicine, the Olatunde Odeku Medical Library, was not functional at the time of this study because the building that accommodates it was under physical expansion. The theme also revealed inadequate hostel accommodations and facilities. The reason for this is that there was not enough room to accommodate all clinical students in Alexandra Brown Hall, the only hostel for clinical students. The authorities of the College of Medicine and the University of Ibadan may have to look into the possibility of providing more rooms or a second hostel for clinical students. Additional problems include the restriction of the use of certain electrical appliances that make life easier, frequent lack of water supply, and even harassment of clinical students by hostel porters.

Physiotherapy students rated the percentage of students’ perception of teachers (SPOT) second, while nursing students rated the percentage of students’ perception of learning (SPOL) second out of the five domains of DREEM. Despite rating these second, the perceived teaching methods were unsatisfactory. The theme “perception of teaching method” enumerated unsatisfactory perceptions of teaching methods and poor communication skills by lecturers. This could be because most of the lecturers had no formal training in teaching methods before becoming lecturers. Anecdotal evidence suggests that a small proportion of lecturers in colleges have formal training as teachers. Academic or professional postgraduate programs generally lack teaching methods. However, it is gratifying to find out that the College of Medicine started running a master’s degree in biomedical education a few years ago. Research indicates that lecturers who receive formal training in pedagogical approaches maintain more positive lecturer-student relationships within their learning environments [53, 54]. Therefore, all young lecturers (those below senior lecturer grade) can be sponsored to participate in this program, and experienced (those with grades equal to or above senior lecturer grade) individuals can attend regular biomedical education workshops to improve their teaching methods. This approach will improve teaching methods and feedback mechanisms from lecturers to students. Based on the findings of this study, some lecturers were perceived not to provide a conducive atmosphere for students to interact well with them and discuss things even outside their academic topics. Such students may be starved of emotional stability a lot of the time and thus may not feel excited about their education. The University of Ibadan will do well to empower its academic staff to improve these aspects. The theme ‘perception of curriculum/course content’ highlighted that the curricula are bulky, with very little time allocated to covering the course content. This could be partly due to the fact that dental and medical students are the first individuals to use revised dental and medical curricula, which they might have compared to the old curriculum. A previous study suggested that this might be due to a slower evolution of the hidden curriculum, which masks the gains of the innovative curriculum [43]. The students also complained of unduly long hours spent in school per day. Sometimes the students do not even know when they would get back from school, for example, in surgery postings. This has thus adversely affected their social life. This observation was similar to that of a previous study suggesting that students were dissatisfied with the school timetable [39].

Although the results of the quantitative surveys for all the programs showed more positive perceptions than negative perceptions, the differences in the perceptions of the learning environment across the programs may indicate strengths within certain programs rather than weaknesses within others. For instance, the physiotherapy program had higher overall DREEM and SASP scores than did the other programs, while the overall DREEM and SSSP scores were the lowest for the medicine program. The weaknesses were identified by the qualitative aspects of the results. Previous studies among health sciences students have also identified similar significant differences in the perception of the learning environment across the programs [10, 31, 33].

The learning environment of students in Nigeria has been strongly affected by many factors, including incessant strike actions, lack of understanding of the curriculum by both lecturers and students, inadequate hostel accommodations and uncomfortable classroom environments, absence of cordial lecturer-student relationships, and lack of equipment. Teachers should aim to provide an environment in which students feel free to voice their concerns, identify their lack of knowledge, and stretch their limits [8]. It was reported in a study that teachers not only teach but also perform many other nonteaching tasks and functions (i.e., administrator, counselor, and friend) [55]. It is worth noting that the students who participated in the qualitative study expressed more negative perceptions of their learning environment. This apparent discrepancy between the results of the quantitative study, which revealed more positive than negative perceptions of the learning environment and the results of the focus group discussions which revealed negative perception, might be because the students had the opportunity to express these negative perceptions during the focus group discussions. This is unsurprising, as the mixed-methods approach offers greater insight that single method approach may not provide.

The learning environment is not limited to lecturer-student interactions, teaching, and learning activities; it also includes having good physical structures and facilities provided by the institution [6]. These include good and adequate chairs, sufficient classrooms, well-equipped and easily accessible libraries, well-ventilated classrooms, regular electricity supplies, technical knowledge, and available equipment for learning. A social learning environment equips learners with the tools necessary to collaborate and participate with teachers and peers both inside the classroom and beyond the walls of the school. A safe social learning environment can in effect extend the relationship among students and allow continued dialog and collaboration beyond school hours [12].

This study complements existing literature to understand better clinical students’ perception of LE in the context of Nigeria. While the previous studies in Nigeria assessed LE of a single program [25, 32, 39–44], the current study added to the understanding of LE among clinical students by reporting students’ perceptions and comparing four programs. The students’ experiences of LE elaborated on the negative perceptions reported among the clinical students. There is a need to strive to provide optimal experiences for clinical students in curricula review, implementation, and teaching.

The strength of this study is the mixed-method research approach. The qualitative aspect of this study provided further insight into the students’ experiences of their learning environment, especially the negative experiences reported. However, the results of this study should be interpreted with caution, as our findings are context specific. Therefore, context should be considered when the generalizability of our findings to other student cohorts and institutions is considered.

## Conclusion

Clinical students have more positive than negative perceptions of their learning environment. Students’ self-academic perceptions, student-centered learning and teachers’ knowledge base are perceived as positive, while student-lecturer relationships, poor infrastructures and bulky curricula with resultant restricted social life are negative experiences reported. Efforts should be directed at expanding and improving infrastructure facilities to improve the learning environment. Other critical areas perceived as negative should be addressed by the authorities. Future studies may look at the influence of the learning environment on students’ performance at various levels of study across clinical programs in the institution.

### Electronic supplementary material

Below is the link to the electronic supplementary material.

Supplementary Material 1

## Data Availability

The datasets analysed during the current study are available from the corresponding author on reasonable request.

## Abbreviations

LE: Learning environment
DREEM: Dundee Ready Education Environment Measure
MBBS: Bachelor of Medicine and Surgery
BDS: Bachelor of Dental Surgery
BNS: Bachelor of Nursing Science
B. Physio: Bachelor of Physiotherapy
BMLS: Bachelor of Medical Laboratory Science
SPOL: students’ perceptions of learning
SPOT: students’ perceptions of lecturers
SASP: students’ academic self-perceptions
SPOA: students’ perceptions of the atmosphere
SSSP: students’ social self-perception
