# Supplementary material for: Clinical students’ perception of educational environment in a Nigerian university: a mixed method study
Source: BMC Med Educ. 2024 Jul 4;24:725. doi: 10.1186/s12909-024-05734-2 (PMC11225165; doi:10.1186/s12909-024-05734-2)
Supplement: Supplementary file 1 — Supplementary Material 1 [file 12909_2024_5734_MOESM1_ESM.docx]

**Appendix 1**

**THE FOCUS DISCUSSION GUIDE QUESTIONS AND PROBES**

**Question 1:** What are some of your challenges in the learning process?

**Probe:** Is the teaching student centered? Are course objectives provided at the start of the course? Is teaching teacher centered? Is the teaching period put to good use? Does teaching emphasize the long term more than the short term?

**Question 2:** What can you say about the whole teaching process in your discipline (dentistry, medicine, nursing, physiotherapy)?

**Probe:** Are the teachers knowledgeable? (Method of teaching, lecturer-student relationship, and teachers’ organization, preparation and feedback).

**Question 3:** What are your views on your learning environment?

**Probe:** How conducive is it to learning? (Infrastructure, facilities, library, equipment).

**Question 4:** Does studying your course (dentistry, medicine, nursing, physiotherapy) have any influence on your social life? How?

**Probe:** Does studying your course (dentistry, medicine, nursing, physiotherapy) restrict you from or expose you to social life? Do you have time for other things apart from academic activities? How is your relationship with lecturers and students? How often do you make new friends?

**Question 5:** Is there anything more you would like to share with regard to your learning environment?
